# St. Louis Medical Society

**Published:** 1858-03

**Authors:** 


### SELECTIONS.

### *St. Louis Medical Society.*

DR. MCGINTIE, President, in the Chair.

Reported by L. CH. BOILLINIERE, M. D., Secretary.

DR. POPE related to the Society a successful case of

### OVARIOTOMY.

He had seen the patient only a few days before the operation. She has been married eighteen months and is the mother of a child; the tumor existed, however, before her marriage, and she had been tapped twice, the last time six weeks before the operation. When he saw her, she was bed-ridden, and presented a marked degree of emaciation—there was also anasarca. He saw her then in consultation with Dr. M. M. Pallen.

Dr. Pope, taking in consideration all the circumstances of this case, thought it favorable for an operation.

The patient, although informed of the uncertainty of the prognosis in her case, manifested a rare degree of fortitude. The operation was performed on the first of November, now nearly two months. Prior to proceeding to it, a purgative dose of oil was administered, and on the day of the operation she had her bowels evacuated by an enema. This precaution he considered very important, as the bowels were afterwards to be kept perfectly quiet for a few days by opiates.

She was placed on a table with her legs hanging over the side of it, and brought under the full effect of chloroform. An incision five inches in length, was first carried along the linea alba down to the peritoneum; this was found agglutinated to the external layer of the sac, the interior of this sac having been reached, a bloody, claret-color fluid escaped, which did not however penetrate into the peritoneal cavity on account of the above named adhesions. The incision was then extended two inches more, and a finger introduced in the sac, with the view of drawing it out through the external opening. This sac was, however, found everywhere adherent. These adhesions arose from prior inflammation which had attacked the peritoneum at different points during the course of the patient's illness. By using a certain amount of force, the adhesions were at length broken up and the sac extracted. It was at first thought to be connected with the right ovary and not with the left—the hand could pass all around it on the left side. On that side, a portion of the tumor presented in color the appearance of containing some fœcal-looking matter; but on closer examination, this was recognized as being an additional tumor. The peduncle was twisted twice on itself, and this accounts for the tumor being found on the right side; by twisting upon its base, it had in fact inclined to the right. By this twisting, the peduncle itself was made smaller and harder. During the course of this operation, the bowels were seen only once, they were quiescent when seen. This was undoubtedly to be attributed to the effect of chloroform, and may be considered as a remarkable illustration of its effect— anæsthesia having extended even to the viscera of organic life. A ligature of strong thread was then thrown around the base of the peduncle, and this was drawn out through the external opening.

The next step in the operation was the closing of the opening. This was done by pains and the twisted suture, using for this suture the remainder of the thread used in securing the peduncle. It has been noticed before, that the hanging in the abdominal cavity of the ends of the thread used to tie the peduncle had frequently proved a source of inflammation, perhaps of failure. Opium was given for fourteen days in order to quiet the peristaltic action of the bowels, and the urine drawn three times a day. This precaution he considered very important. Patient was kept on barley water and ice.

About the fourth day a profuse discharge of a bloody, fetid pus occurred. Union by the first intention had then pretty generally taken place. This discharge continued for a few days, and it was not before the twelfth day that healthy pus began to be discharged. The pins were removed on the sixth day. The patient's recovery may be now considered entire.

Dr. Pope would add, that this operation, although formidable, is not so dangerous, in his opinion, as formerly thought. The opening of the abdominal cavity has been by more modern statistics, proven not to be more fatal than the capital operations, such as amputations of the thigh, the ligature of large arteries, etc.

DR. MONTROSE A. PALLEN reported a case of gonorrhœal ophthalmia, followed by the most serious results, producing in the right eye a complete opacity of the cornea, complicated with a staphyloma of the iris, occupying the superior third; upon the left eye there was a vascular opacity of the cornea, adhesions of the iris to the cornea, (synechia anterior,)

and an almost total obliteration of the pupil. The patient was able to distinguish light in both eyes, rendered completely helpless by the accident, having to be lead about by his mother. The manner in which he contracted the gonorrhœal ophthalmia was a very common one among certain classes of people, viz., that of bathing the eyes in urine, in order to strengthen the sight: the patient in question performed this vulgar action whilst laboring under an acute attack of gonorrhœa; the results were as described above; and Dr. Pallen thought that an operation on the right eye was not justifiable as the condition of the parts was not such as to warrant it, at least, with any hope of success. Upon the left was performed that operation of artificial pupil, technically known as *Kerato-Iridectomia*, or the method of Cheselden, modified by Desmarres: this consists in making an incision in the cornea, one-half of a line from the sclerotica and parallel to it; then breaking up any adhesions which may exist, by an instrument (a curette) adapted to the purpose, (very extensive adhesions existed in the case in question,) seizing the iris with a rat-toothed forceps drawing a sufficient portion through the wound, and then snipping it off with the curved scissors. The blood flowing freely from the vessels of the iris, obscured what was revealed after its absorption, viz., a cataract which is of the capsulo-lenticular variety, evidently very hard, and which will be extracted as soon as the eye will have recovered sufficient tone to bear a second operation. The operation for the artificial pupil terminated with all of the success to be desired; the only plan of treatment was rest, confinement to a dark room, and ice kept constantly to the eye.

With regard to the propriety of the operation for artificial pupil, Dr. P. continued, there could be no doubt, and since its first proposal by Cheselden in 1727, it had been practised with varied success, by both of the Wenzels, Scarpa, Beer, Jager, Arlt, Græfe, Mackenzie, Desmarres, and a host of others. To Cheselden, however, is due the honor of having first drawn the attention of the profession to this most interesting branch of ophthalmology; and he alone devised the three original kinds of operation, viz., by *separation*, *incision* and *excision*, which have been modified in various ways by different ophthalmologists; these operations are known as *coredialysis*, *corectomia*, *iridotomia*, *iridectomia*, *keratectomia*, *kerato-iridectomia*, *iridotomedialysis*, and others, according to the whim and the method of the operator.

Dr. P. remarked, that he would not enter into any details with regard to the operation, or any rules concerning it, knowing that he could not do justice to it without detaining the Society beyond the limits of a mere report of a case, and should he do so, he would tax the patience of the gentlemen present; neither would he discuss the merits of the different methods, of separation, of incision, or of excision, or detail the conditions necessary for the operation, but simply state that he preferred the method of excision, such as practised upon the case in question, as offering the best chances for success, as attended with a better result, and as being the most surgical.

Dr. Pallen also related a case of an anterior sub-luxation of the humerus, the head of the bone resting partially upon the outer margin of the pectoralis major: it was of six weeks' standing, and a reduction by the pulleys had been attempted in Alton some two weeks previously unattended with success. Dr. P. succeeded in reducing the bone by the old Hippocratic method, the precepts of which are so well indicated by Malgaigne, viz: by forcible extension, sudden flexion and rotation, thus throwing the head of the bone into the glenoid cavity.

DR. W. M. MCPHEETERS reported to the Society a case of

UNUSUAL RIGIDITY OF THE PERINEUM.

This case, said he, was interesting in many respects, especially in reference to the obstetrical procedure which was resorted to for its relief.

At 9 o'clock, A. M., he was called to a lady then in labor with the first child. Things had proceeded on slowly but regularly, and in the evening the child's head had fairly descended into the pelvic cavity; at midnight, the head reached the perineum, which it considerably distended; the pains then were so frequent and so powerful, the distension of the perineum was such that at every moment he expected to see the issue of the child's head. However, notwithstanding the energetic action of the uterus, continued without intermission, no progress was perceptible. Fearing at this juncture an imminent and extensive rupture of the perineum, he had recourse to venesection to the amount of thirty ounces, to the warm bath, to fomentations, etc.; the state of nausea in which the patient was for several hours, contra-indicated the use of antimonials. In spite of all these means, the rigidity of the perineum gave no signs of yielding, and in order to deliver the patient he feared that no alternative was then left to him, except to lessen the child's head. However, before resorting to this extreme measure, he thought that he would try another means. It was to incise the perineum. This he did by introducing a guarded bistoury at the margin of the extended vulva and performing an incision in the perineum in a diagonal direction, sufficient in length to procure the desired opening; the opening was hardly completed, when, with strong expulsive pains, the child's head made its appearance externally, and was soon followed by the shoulders and the rest of the body. He thought this result very encouraging, and although not aware that this operation had been performed before, he would certainly have recourse to it again under the same circumstances, and would recommend it to others.

He would further remark, that the child was born alive and that the patient recovered without any untoward symptom.

Dr. Pallen said, that in his opinion, Dr. McPheeters, under the above circumstances, had adopted a most judicious course of conduct. Not only was the operation warrantable, but it had been recommended by the highest, such as Dr. Blundell He had himself taught this procedure and applied it in similar cases for many years. Lacerations of the perineum were, said he, very much to be deplored and should be prevented with the utmost care. This laceration may extend sometimes very far, involving the sphincter ani, etc. However, in a case of that description, he had observed an admirable proviso of nature, which consisted in the establishment of another internal sphincter for the anus above the seat of laceration, thus preventing the disastrous consequences of this lesion under ordinary circumstances.

Dr. Hammer, in reference to the case related by Dr.McPheeters, said, that the procedure he adopted in this instance was a regular obstetrical operation taught and practiced in Germany for many years. Naegele, whom he heard lecturing on this very subject, speaks of this operation as having been of long use in Germany. Dr. Hammer added, that in order to make the incision fulfil its object which was the enlargement of the vula, the incision should be sometimes rather extensive and deep. If the incision, said he, was not sufficient, the pressure of the child's head would certainly continue it and produce thus a ragged cut instead of the clean incision which the knife would perforn. He would remark, however, that it is a thing of rare occurrence that the distension of the perineum be so great as to necessitate this operation. By referring to the statistics of the large hospitals, we seldom notice this operation mentioned. In Germany, forceps are generally used in such cases. Their action is to alter the form of the child's head, by elongating it and thus facilitating its passage. Moreover, lessening the rigidity of the perineal muscles by other means; among these, chloroform by inhalations or local fomentations. Kneading the perineum, he said, would also often be of benefit. Killian, of Bonn, recommends this method.

Dr. Pallen, in reply to the above remark, said, that he thought that the application of the forceps would have here increased instead of diminishing the difficulties. The forceps, said he, are compressors of the head only to a very limited degree, as shown by Baudelocque. The forceps is mainly an instrument for extraction, a *tire-tete.* Chloroform, said he, was advisable here for many reasons. It does relieve spasmodic muscular action; he had tried it many times with marked success in case where the version was indicated, and where it would have been next to impossible without the relaxing influence of this powerful anæsthetic.—The kneading, spoken of, merits also, said he, commendation; he had practised many years this method, which he once learned from Dr. Linton.

DR. MCPHEETERS replied, that he was well convinced that in this case an application of the forceps would have inevitably caused a rupture of the perineum. He had often applied them under similar circumstances, but not when there was that degree of rigidity; he added, that fomentations and inunctions of oil had been freely used; the patient was already naturally nauseated so as to preclude the use of antimony, she had been bled to a considerable amount, and the co-aptation of the head to the perineum was so intimate that the very act of introducing the forceps would have produced laceration. Furthermore, there were expulsive pains enough without the forceps. Chloroform was not administered on account of the repugnance of the patient's friends to it. Morever he considered that the very pressure of the child's head was the greatest relaxant that could have been devised; and all the above means, after a fair trial, proved of no avail. But after the incision made in the perineum, the child's head issued without the slightest difficulty.

DR. HAMMER considered the variety of opinion regarding the use of the forceps, in these cases, as a mere matter of discrepancy between two continental schools. In England and America, craniotomy, said he, was outrageously frequent whilst in France and Germany forceps operations were preferred to the former more bloody procedure. The Germans consider that a certain degree of disproportion between the child's head and the pelvis or the perineum may be overcome by a judicious use of the forceps. However, the disproportion is sometimes so great that it can not be overcome. Under the circumstances of Dr. McPheeter's case, the Germans would have used the forceps; there was, it is said, an impossibility of introducing the instrument during the pains, but in the absence of a pain this might have been done. It has been said that the rigidity of the perineum is principally kept up by the presence of the child's head; but the spasmodic contractions of the perineum thus induced, alternate with those of the uterus. By the action of the forceps this alternation is annulled by the continued pressure it allows to be made on the perineum with the child's head, whilst it is embraced in the blades of the forceps.

Naegele, although of a timid temperament, in other things, used the forceps liberally.

The forceps, by compressing the child's head, elongated it. This instrument did not perhaps much diminish the size of the child's head, but by altering its shape it facilitated its passage through a narrow pelvis. Did not nature proceed in the same manner in cases of disproportion between the head and the pelvis? In those cases it was to be remarked that the head was sometimes considerably elongated. In this wise did the action of the forceps imitate the operation of nature.

DR. MCPHEETERS admitted the possibility of this elongation. As for using the forceps in this case, he considered it was utterly impossible either during a pain or in its absence; for the fitting of the head to perineum was so close the patient complained loudly of the mere introduction of the finger, which she compared to a cutting instrument.

As to the spasmodic contractiliy of the perineum referred to by Dr. Hammer, it was altogether destroyed in this case, as will occur whenever the muscular fibre has been acting powerfully for a length of time.

DR. M. M. PALLEN thought that so far as this case was concerned, the delay of the passage of the child's head through the vulva was not owing in any degree to inertia of the uterus or to a want of the *vis a tergo*; this existed to a sufficient extent to have procured the birth of the child under ordinary circumstances; but here was a most rigid perineum, against which the child battered in vain—a danger of laceration was imminent as the result of natural forces alone—the forceps, he thought, would have increased the danger of this laceration. A great deal of prudence should guide the accouchenr in deciding upon the cases where the forceps would be useful. He was himself a strong advocate of the forceps under certain circumstances, although he must say that he condemned the use of the *long* forceps under any circumstances. In this, he was aware that he differed from the opinions taught by all accoucheurs even in America, and especially in Germany. He was not, however, inclined to undervalue the great and valuable progress made in the art of obstetrics by the Germans; he knew full well that Naegele had done more than any man, except Baudeloque, to advance this art. But whilst he acknowledged their eminence he did not in everything adopt the teaching of that school; he endorsed the doctrines taught in London and Dublin; he was convinced from statistics, that more women recovered after craniotomy than after the use of forceps when applied above the pelvis. The mortality of the children was also very great in those cases; frequently, also, various ruptures, and also vesico or recto vaginal fistulæ were the result of the imprudent applications of the forceps. Siebold had, said he, applied the forceps once in every seven cases of midwifery which occurred in his practice. This preference of the Germans for the forceps was, he said, not based upon merely scientific reasons, but resulted in a great degree from peculiar religious prejudices.

DR. HAMMER, in reply, denied that the German accoucheurs were influenced by peculiar religious opinions in this matter; their adoption of the forceps, in many cases where it was not employed by the English, was based upon purely scientific grounds. In Germany, the forceps was better studied and understood in all its applications, and selected therefore as a very safe and useful instrument in cases where the English or American accoucheur would, without hesitation, sacrifice the child's life In Germany, in those cases, they tried to save the child and the mother also, and they generally succeeded. In England, one of the reasons why the forceps were not used was on account of that m .ck modesty so peculiarly Briti-h, which did not allow of a woman to be uncovered. Acting on this false modesty they have not encouraged the establisment of lying-in hospitals to the same extent that they have done on the continent.—They do not understand the forceps for want of facilities to study its applications, hence their hesitation in applying it. The Germans, who are familiar with that instrument, are more bold in its employment.

As for the action of the forceps in the case of Dr. McPheeters, he thought that the rigidity of the perineum was owing to a spasmodic contraction of the muscles of that part, comparable to the spasmodic stricture which occur sometimes in the urethra. If a bougie is introduced when one of these spasmodic strictures eixsts, it will impinge against it, without penetrating through it; but if the bougie be left there for a few minutes. the parts will gradually become accustomed to it, and it will at length penetrate though the stricture. The same process will occur in spasmodic contraction of the perineum. This said he, was the rationale of the action of the forceps in those cases. If the child's head be pressed for some time by the forceps on a rigid perineum, the spasmodic contraction of that part will gradually yield. As for the ruptures and vesico or recto-vaginal fistulæ, which according to Dr. Pallen, were often the result of the use of the forceps, he thought that they took place oftener when the forceps were not used, as in those cases which have been left to nature or where craniotomy was performed. Vesico-vaginal fistulæ

happen mostly in the protracted cases which had been left to nature.—Statistics would prove this.

In the United States, there are, said he, as many craniotomy cases as there are forceps cases in the rest of the world; because the forceps are not used at all in some cases where it could have been beneficially applied in the beginning of the labor, and craniotomy is resorted to as a last resort. He would not blame the profession here for this state of things; it originated from the want of lying-in hospitals, as he had already remarked.

Dr. Pallen said, that he would see with the greatest satisfaction the establshment of lying-in hospitals in this country, and he expressed the hope that the Biddle Lying-in Hospital, in process of construction in this city, would some day prove a source of practical instruction to students desirous of perfecting themselves in the science of obstetrics. As for himself, he was far from condemning the use of forceps at the inferior strait; he was in the frequent practice of applying it himself—and he had taught its utility for years;—he would add, that his opinion was, that it was not applied in many cases in this country where its usefulness would be incontestible if better understood, and where craniotomy was unfortunately, performed, especially in the country, where the practitioner is often left to himself.

Dr. Smith was one day present, said he, at a lecture on obstetric operations, delivered by Dr. Murphy, of London; in that lecture, he made the startling remark, that statistics would bear him out in the assertion that death oftener followed operations of craniotomy at the superior strait than cæsarean sections.

Dr. Smith said, that as for the rigidity of the perineum giving way under the continued pressure of the child's head, as asserted by Dr. Hammer he would rather expect to see the rigidity increased under this prolonged pressure; he would expect to see inflammation setting in under these circumstances, and a spasmodic contraction firmly established. As for the procedure employed by Dr. McPeeters, in the case he had reported, he thought it perfectly warrantable and worthy of imitation under similar circumstances.—*St. Louis Medical & Surgical Journal.*

